# Process Technologies and Device Applications of Transition Metal Dichalcogenide (TMD)-Based Two-Dimensional Semiconductors: A Review

**DOI:** 10.3390/mi17101153

**Published:** 2026-09-30

**Authors:** Do Hyeon Lim, Sooyeon Kim, Jinsoo Shin, Eunmi Park, Sung Gyu Pyo

**Affiliations:** School of Integrative Engineering, Chung-Ang University, Seoul 06974, Republic of Korealeafgreen0504@cau.ac.kr (J.S.); eunmi5308@cau.ac.kr (E.P.)

**Keywords:** transition metal dichalcogenides, two-dimensional semiconductors, MoS_2_, phase engineering, contact resistance, van der Waals contact, chemical vapor deposition, wafer-scale growth, ultrascaled transistors, monolithic 3D integration

## Abstract

With the continuous miniaturization of semiconductor devices, the limitations of conventional Si-based devices—including short-channel effects, increased leakage current, and elevated power consumption—have become more pronounced, drawing increasing attention to atomically thin two-dimensional (2D) semiconductors as candidate channel materials for next-generation devices. Transition metal dichalcogenides (TMDs) exhibit electronic and optical properties that vary with layer number; in particular, monolayer MoS_2_ possesses a direct bandgap and excellent electrostatic gate control, making it a promising material for ultrascaled transistors and optoelectronic devices. In this review, we examine the structural and electrical properties of TMDs, focusing on phase engineering for crystal-phase control, semimetal electrodes, and van der Waals contacts for reducing contact resistance at metal/TMD interfaces. We further analyze wafer-scale TMD growth via chemical vapor deposition (CVD) and metal–organic CVD (MOCVD), as well as recent progress in epitaxial growth for controlling grain boundaries and growth orientation. From a device perspective, we review TMD transistors with 1 nm and sub-1 nm physical gate lengths and discuss their extension toward high-frequency devices and three-dimensional integration. These findings demonstrate that TMDs are promising channel materials capable of overcoming the scaling limitations of conventional Si-based semiconductors; however, challenges remain for practical implementation, including large-area material uniformity, contact resistance, defect and interface control, process reproducibility, and compatibility with existing CMOS processes.

## 1. Introduction

According to Moore’s Law, the modern semiconductor industry faces fundamental limits in device integration density. As the dimensions of conventional silicon (Si)-based devices are scaled down to the tens- or single-digit-nanometer regime, short-channel effects become increasingly severe [1], and the resulting increase in leakage current and power consumption has become a major obstacle to further improving device performance. To address these technical challenges, extensive research has been conducted on two-dimensional (2D) nanomaterials whose thickness can be controlled at the atomic level.

The physical origin of this scaling difficulty lies in electrostatics: as the channel length of a bulk-like Si transistor is reduced, the gate progressively loses electrostatic control over the channel potential relative to the source and drain electrodes. This gives rise to drain-induced barrier lowering (DIBL), a degraded subthreshold swing, and an increase in off-state leakage current, all of which erode the power and performance benefits that scaling is intended to deliver [2,3]. A widely adopted strategy to restore electrostatic control is to thin the channel body itself, since a thinner channel is intrinsically less susceptible to drain-field penetration. However, ultrathin Si and III–V channels suffer from surface roughness scattering and dangling-bond-related interface states once their thickness approaches only a few nanometers, which degrades carrier mobility and limits how far this approach can be pushed [4]. Atomically thin 2D semiconductors, whose surfaces are intrinsically free of dangling bonds, offer a natural solution to this dilemma, motivating the extensive research effort into 2D-material-based transistors over the past decade.

**Figure 1:**
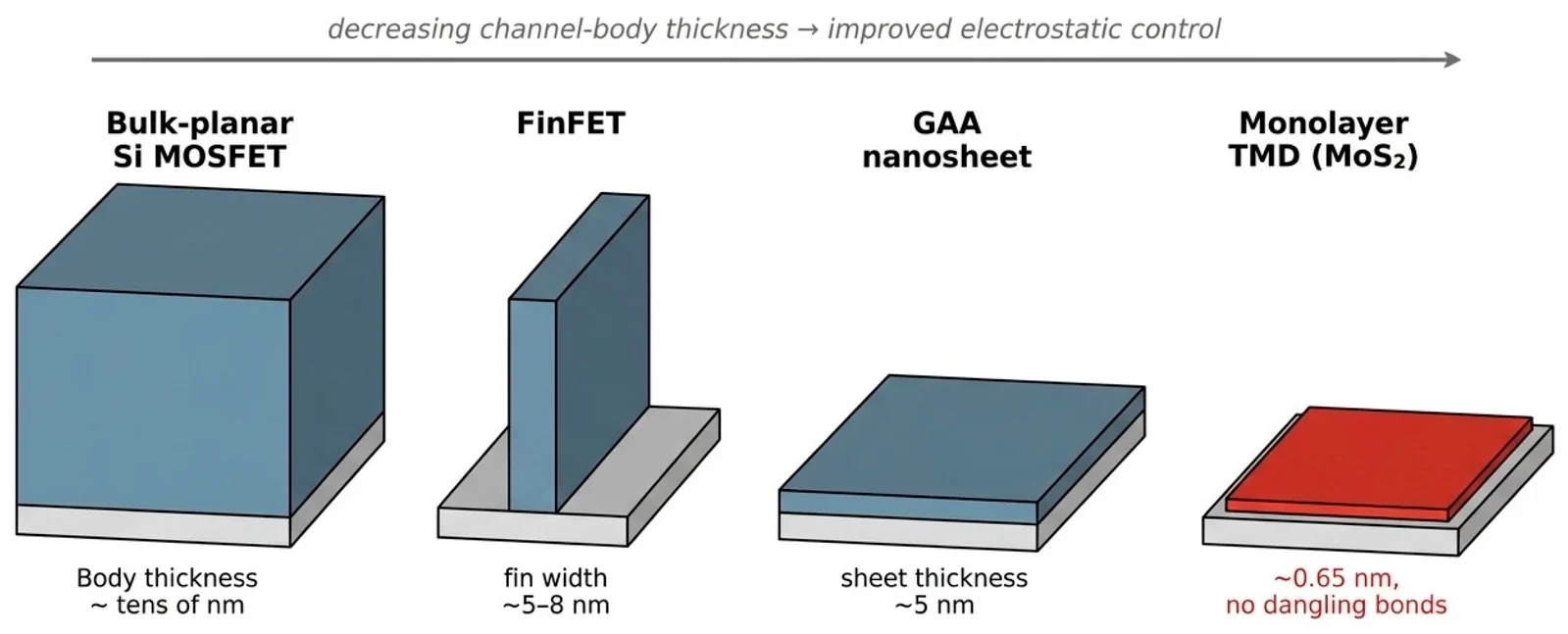
Schematic comparison of channel-body thickness across Si-based device generations and monolayer TMD. Unlike Si- or III–V-channel thinning, monolayer TMD reaches sub-nanometer thickness without a dangling-bond-related mobility penalty, since its surface is intrinsically free of dangling bonds.

For comparison, satisfying this same electrostatic-scaling criterion in a Si- or III–V-channel platform requires the fin or nanosheet body thickness to be reduced to only a few nanometers at advanced technology nodes; at such dimensions, surface-roughness scattering and dangling-bond-related interface states impose an intrinsic mobility ceiling that cannot be removed by further process optimization [4]. TMDs largely sidestep this trade-off: because a monolayer is already only ~0.65 nm thick and its surface is intrinsically free of dangling bonds, comparable electrostatic control can be obtained without incurring the mobility penalty that ultimately limits how far Si- or III–V-channel thinning can be pushed (Figure 1).

Among 2D materials, graphene was the first to attract broad attention due to its exceptionally high carrier mobility; however, its lack of an intrinsic bandgap results in a poor on/off current ratio, limiting its usefulness for digital logic switches despite its attractiveness for high-frequency analog applications [4]. This limitation has driven interest toward semiconducting 2D materials with a sizable, tunable bandgap, among which transition metal dichalcogenides (TMDs) are regarded as one of the most promising next-generation materials [4,5]. In particular, MoS_2_ exhibits an indirect bandgap in its bulk form that transitions to a direct bandgap in the monolayer limit, a material characteristic that offers decisive advantages for realizing high-performance optoelectronic devices and low-power transistors [6,7]. The successful demonstration of a high-performance monolayer MoS_2_-based field-effect transistor (FET) using a high-k gate dielectric has already provided experimental evidence for the feasibility of practical 2D-material-based devices [8].

Nevertheless, several key challenges remain to be addressed before TMDs can be integrated into practical semiconductor manufacturing processes. The high contact resistance and Schottky barrier that arise at the interface between metal electrodes and 2D materials are major factors limiting device mobility and switching speed [5,9]. To overcome these limitations, innovative process technologies—including van der Waals (vdW) contact formation, phase engineering to control the 2H–1T phase transition, and the introduction of semimetal electrodes—have been proposed to minimize interfacial resistance [10]. At the same time, because device performance is only as good as the material from which it is fabricated, the ability to grow large-area, uniform, and reproducible TMD films is just as critical as interface engineering; a device concept that works only on exfoliated micrometer-scale flakes has limited relevance to manufacturing.

In this review, we analyze CVD/MOCVD process technologies for large-area deposition and recent research trends in interfacial property enhancement, based on the physical properties of TMD materials [11,12]. We further examine examples of 1 nm-scale advanced process technology and present a technical roadmap for integrating 2D materials into existing CMOS lines, with the aim of assessing the feasibility of realizing next-generation TMD-based semiconductor devices [13,14,15]. Beyond summarizing individual results, we also organize representative reports on contact engineering, large-area growth, and device scaling into comparative tables, and we conclude with a dedicated discussion of the remaining challenges—material uniformity and metrology, contact resistance scaling limits, dielectric integration and reliability, and manufacturing compatibility—that must be addressed before TMDs can move from laboratory demonstrations to industrial adoption.

## 2. TMD Materials and Process Technologies for Semiconductor Applications

### 2.1. Structural and Electrical Properties of TMDs

**Figure 2:**
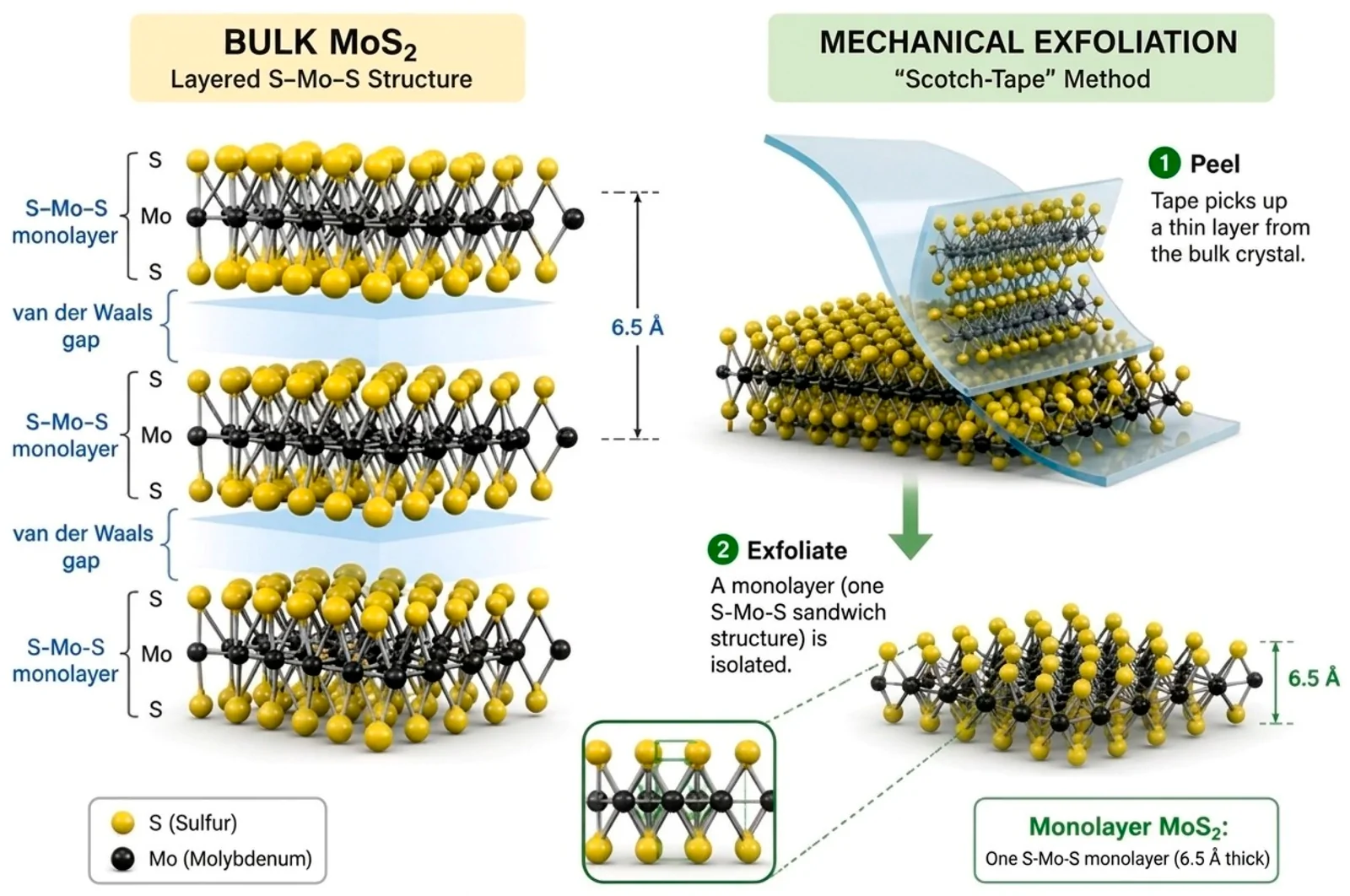
Original schematic illustration of the layered structure of bulk MoS_2_, showing van der Waals gaps between adjacent monolayers (each an S–Mo–S sandwich) and the mechanical (“scotch-tape”) exfoliation of a single 6.5-Å-thick monolayer [8].

Transition metal dichalcogenides (TMDs) are layered materials represented by the chemical formula MX_2_, where M denotes a transition metal (e.g., Mo, W) and X denotes a chalcogen element (e.g., S, Se, Te). A single TMD layer has an X-M-X atomic arrangement, in which a layer of transition-metal atoms is sandwiched between two layers of chalcogen atoms. Strong covalent bonding exists within each layer, whereas adjacent layers are held together by weak van der Waals forces (Figure 2). This bonding structure allows TMDs to form atomically smooth surfaces, providing a 2D interface with a low density of dangling bonds from a solid-state physics perspective [4].

One of the representative physical properties of TMDs is the layer-number-dependent variation in electronic band structure. MoS_2_, a representative TMD material, has an indirect bandgap of approximately 1.29 eV in its bulk form [16,17]. However, as the material thickness is reduced to a monolayer, changes in interlayer interaction and electronic structure give rise to a direct bandgap of approximately 1.8–1.9 eV [5,6,18]. According to Mak et al., as MoS_2_ is thinned from bulk to monolayer, the indirect bandgap increases by more than about 0.6 eV, whereas the direct bandgap at the K-point shows only a minor change of about 0.1 eV. As a result, in the monolayer limit, the indirect bandgap is pushed to a higher energy level than the direct bandgap, resulting in a crossover in the electronic band structure.

**Figure 3:**
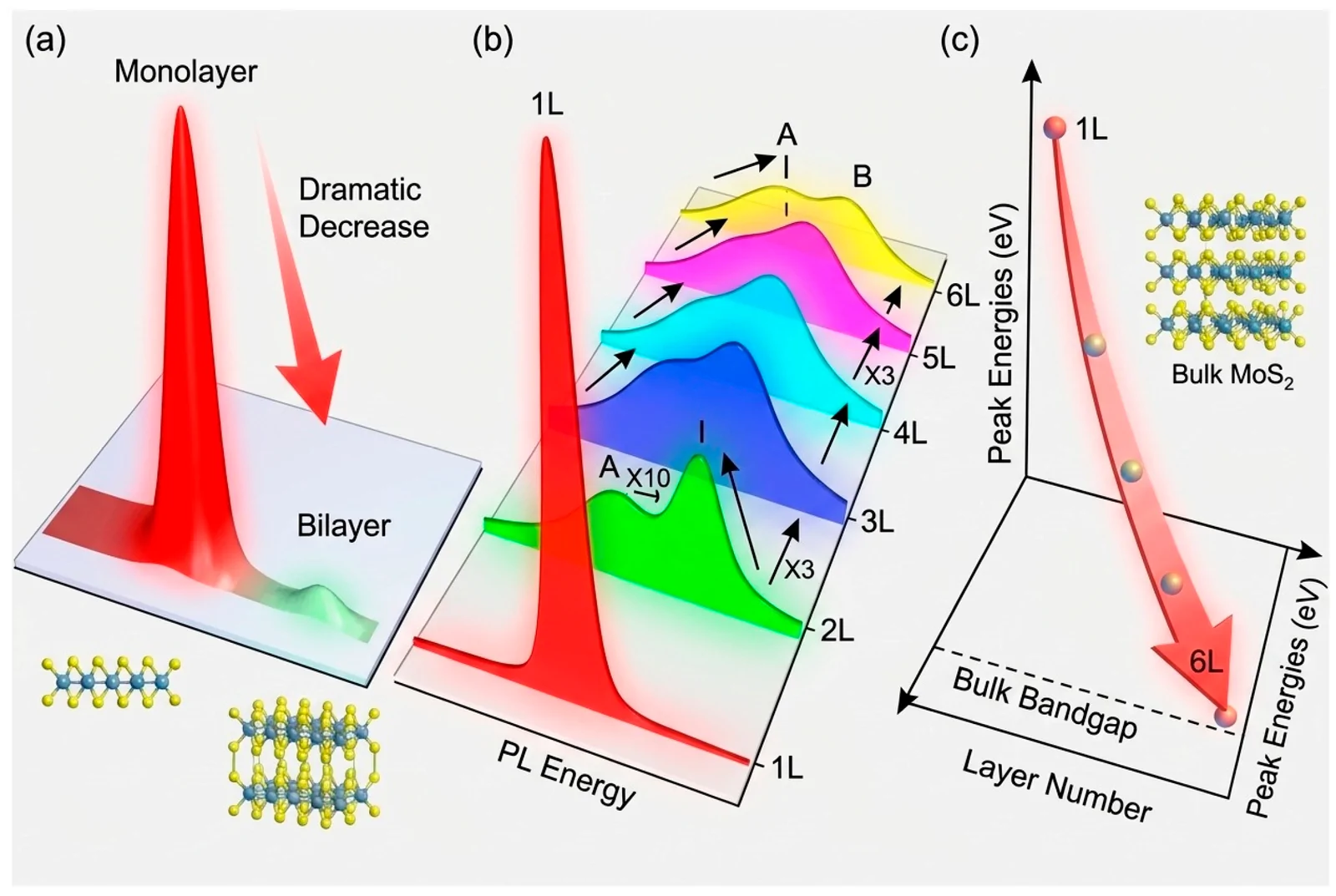
Illustrative schematic of layer-dependent photoluminescence (PL) trends in MoS_2_. (**a**) Qualitative comparison of PL intensity between monolayer and bilayer samples. (**b**) Schematic evolution of PL spectra with number of layers. (**c**) Schematic trend of bandgap energy as a function of layer number, concept adapted from [6].

This change in band structure directly affects not only the bandgap energy but also the optical properties, which can be confirmed through photoluminescence (PL) analysis (Figure 3). Monolayer MoS_2_ exhibits a strong A-exciton PL peak near 1.9 eV, whereas the PL intensity decreases substantially in bilayer and thicker structures. This behavior arises because monolayer MoS_2_ possesses a direct bandgap, while thicker structures exhibit indirect-bandgap characteristics [5,6]. The A and B exciton peaks observed in the PL spectrum are associated with direct transitions near the K-point, and the energy difference between the two peaks mainly originates from valence-band spin–orbit splitting. This direct transition energy shows relatively little variation with layer number [6,7,19]. In contrast, bilayer and thicker MoS_2_ exhibit a lower-energy PL signal originating from the indirect bandgap, and the indirect bandgap energy decreases with increasing layer number due to enhanced interlayer interaction [6,19]. Consequently, in monolayer MoS_2_, the direct transition at the K-point becomes the lowest-energy transition, allowing electrons and holes to recombine directly without requiring an additional phonon-assisted momentum change. This is why monolayer MoS_2_ exhibits stronger PL than multilayer structures [5,6].

In addition, monolayer MoS_2_ can form not only excitons—bound states of electrons and holes arising from strong Coulombic interaction—but also trions, in which an additional free electron binds to the exciton to form a charged quasiparticle, with a reported binding energy of approximately 20 meV [20]. This strong excitonic effect is observed not only in MoS_2_ but also in other TMD materials; for example, a large exciton binding energy of approximately 0.32 eV has been reported in monolayer WS_2_, reflecting the strong electron–hole interaction characteristic of atomically thin 2D TMDs [21]. These optical properties support the potential for optically controlling TMDs via carrier concentration [6,22].

The electronic structure of TMDs is also highly sensitive to external strain, in addition to layer number. In experiments applying uniaxial tensile strain to monolayer MoS_2_, the optical bandgap was observed to decrease at a rate of approximately 45 meV/% with increasing strain, demonstrating that the band structure of TMDs can be mechanically tuned [23,24].

The layer-number dependence also extends to the lattice vibrational properties of MoS_2_, which is exploited in current research. In Raman spectroscopy, the E^1^_29_ and A_19_ modes shift in opposite directions with layer number, and the frequency difference between these two modes serves as a widely used indicator for determining the number of layers in single- and few-layer MoS_2_ [25,26].

Beyond these structural and optical properties, carrier mobility is another critical factor to consider when using TMDs as transistor channel materials. Although monolayer TMDs possess atomic-scale thickness, they can exhibit meaningful carrier mobility through appropriate power management and interfacial engineering. Theoretical studies have predicted a room-temperature intrinsic phonon-limited mobility of approximately 410 cm^2^ V^−1^ s^−1^ for monolayer MoS_2_, while in real devices, transport properties are also affected by charged-impurity and dielectric-environment scattering in addition to electron–phonon scattering [27,28].

In an early study employing a high-k gate dielectric, a field-effect mobility exceeding 200 cm^2^ V^−1^ s^−1^ was reported for a monolayer MoS_2_ FET, demonstrating the feasibility of using TMDs as transistor channel materials [8].

It is worth noting that the experimentally reported field-effect mobility of monolayer MoS_2_ remains well below the theoretically predicted phonon-limited intrinsic mobility of approximately 410 cm^2^ V^−1^ s^−1^ [27,28]. This gap is generally attributed to extrinsic scattering mechanisms that are absent from the ideal theoretical picture, including Coulomb scattering from charged impurities in the substrate or gate dielectric, remote phonon scattering from the surrounding dielectric environment, and scattering from process-induced defects such as sulfur vacancies. Because mobility directly governs both the on-state drive current and the achievable switching speed of a transistor, narrowing this gap between intrinsic and extrinsic mobility—through substrate engineering, dielectric encapsulation, and defect passivation—remains an important objective for realizing the full performance potential of TMD channels in logic and radio-frequency applications alike.

Bulk TMD crystals have also been extensively characterized by Hall-effect transport measurements. Because adjacent layers are coupled only by weak van der Waals forces, in-plane transport in bulk crystals is dominated by intralayer phonon and charged-impurity scattering, giving room-temperature Hall mobilities that are generally well below the theoretical monolayer phonon limit and that increase sharply as temperature is lowered and phonon scattering is suppressed—a hallmark of a phonon-limited transport regime at room temperature. Because bulk-crystal mobility depends strongly on crystal purity, stoichiometry, and measurement geometry, reported room-temperature values vary considerably between studies and growth sources (on the order of 10–102 cm^2^ V^−1^ s^−1^ in typical 2H-MoS_2_ crystals); the authors will confirm the specific value(s) and citation(s) against their preferred primary source before submission.

Table 1 collects the key structural, optical, and electrical property values discussed in this section for bulk and monolayer MoS_2_, providing a compact reference point for the layer-dependent behavior that motivates much of the device work reviewed later in this article.

### 2.2. Crystal Structure and Phase Engineering

**Figure 4:**
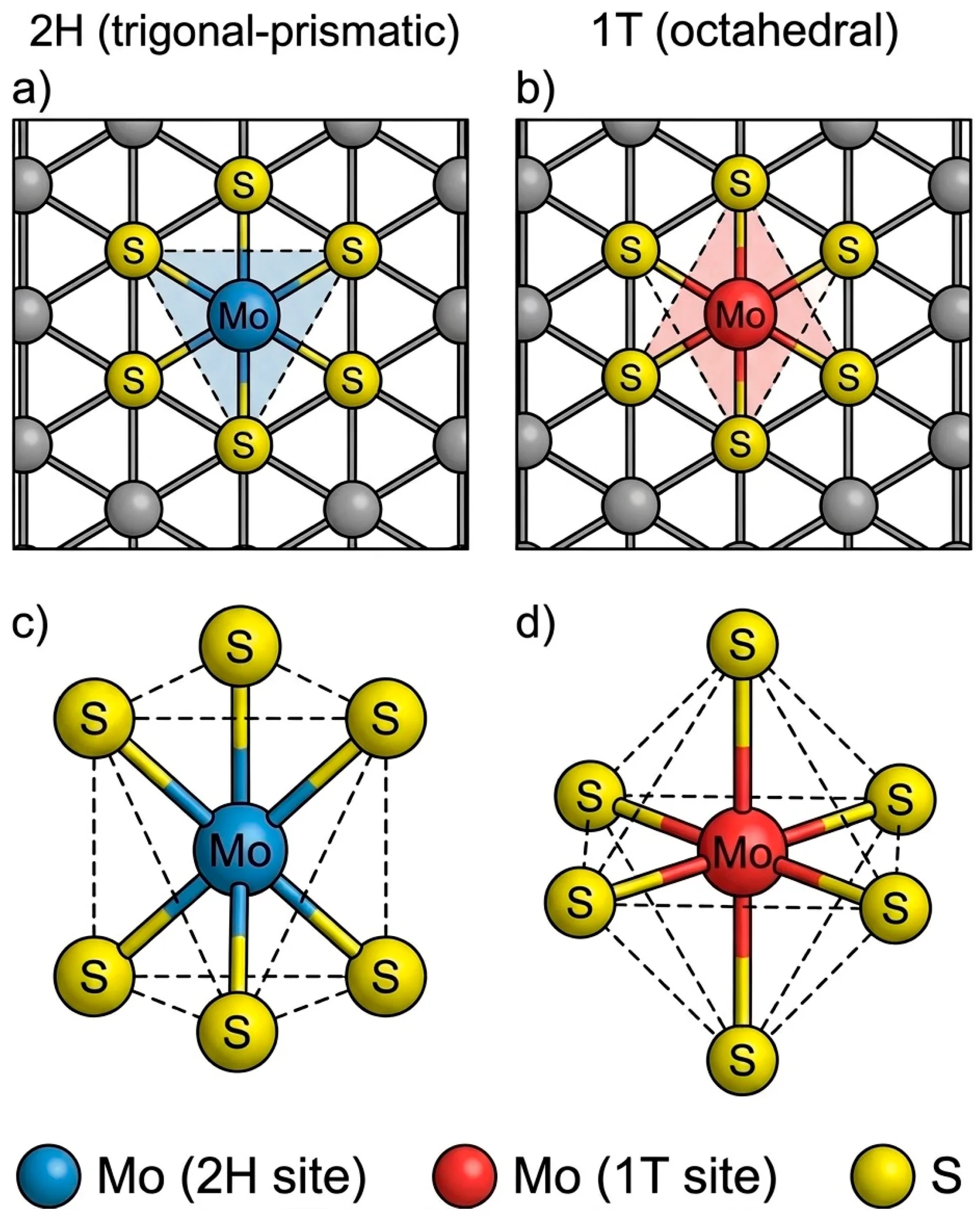
Schematic illustration of the two locally distinct atomic coordination environments reported for monolayer MoS_2_—a trigonal-prismatic (2H-like) arrangement and an octahedral (1T-like) arrangement—consistent with the coexistence of distinct crystal phases within a single nanosheet, concept adapted from [34,35]. (**a**,**b**) Original schematic top-down atomic-arrangement diagrams illustrating the 2H and 1T local registries. (**c**,**d**) Idealized ball-and-stick schematics of the corresponding trigonal-prismatic (2H) and octahedral (1T) Mo coordination environments.

TMDs form various crystal phases depending on the coordination geometry of the transition metal and chalcogen atoms and the interlayer stacking arrangement. Representative phases include the 1H/2H series, which adopts trigonal-prismatic coordination, and the 1T series, which adopts octahedral coordination. In the case of MoS_2_, the naturally stable 2H phase exhibits semiconducting behavior, whereas the 1T series exhibits metallic conduction [29,30,31]. In the monolayer limit, the 2H structure is referred to as 1H, and group-6 TMDs such as MoS_2_ are generally stable in the trigonal-prismatic structure [29,30,32]. In contrast, the metallic 1T phase possesses high electrical conductivity and can therefore be used to lower contact resistance with electrodes, although it has limited thermodynamic stability. This phase transition affects not only electrical properties but also chemical reactivity; enhanced catalytic activity has been reported for the metallic MoS_2_ phase [33]. Furthermore, scanning transmission electron microscopy (STEM) analysis has confirmed that chemically exfoliated monolayer MoS_2_ can contain coexisting crystal phases with distinct electronic properties within the same nanosheet (Figure 4), forming an atomically coherent heterophase interface [34].

The method of exploiting these electrical differences between crystal phases at the device level is known as phase engineering. A representative approach, n-butyllithium-based Li intercalation, injects electrons into MoS_2_, altering the relative stability between the 2H and metallic phases and inducing a structural transition from the 2H phase to the 1T or 1T′ phase [30,32,36,37,38,39]. Using this approach, local phase engineering has been studied in which the semiconducting channel is maintained in the 2H phase while only the electrode-contact regions are converted to the metallic phase [10]. Electron injection can alter the relative free energy between the 2H and 1T′ phases, and it has been theoretically shown that under sufficient electron-doping conditions, the 1T′ phase can become energetically favorable [38,39]. Forming a semiconducting 2H channel and a metallic contact region within the same TMD in this manner enables the realization of a heterophase homojunction, which can be used to mitigate the high contact resistance typically observed at conventional metal/TMD contacts [10]. Accordingly, transition to the 1T phase can enhance the conductivity of the material, providing a solution that overcomes the limitations of conventional metal-deposition approaches. In addition to post-growth phase-transition treatment, bottom-up phase engineering—in which the desired crystal phase is directly selected during the growth stage—has also been studied. Liu et al. successfully achieved the selective growth of metastable 1T′-MoS_2_ monolayers and 1T′/2H heterophase bilayers using K-assisted CVD [40].

It should be noted that the n-BuLi reagent used in this conventional route is a strong, pyrophoric reductant that is highly sensitive to ambient air and moisture, and the resulting Li-intercalated intermediate can likewise be air- and moisture-sensitive, complicating handling and potentially compromising the long-term stability of the converted phase. To address this limitation, alternative intercalation chemistries have been explored. Molten-metal-assisted intercalation, which exploits capillary infiltration of molten potassium into the MoS_2_ interlayer gap, has been reported to achieve 1T-phase purity exceeding 92% within approximately one hour—substantially faster than conventional Li-intercalation protocols (48–72 h)—together with markedly improved long-term phase stability [41]. Separately, intercalation with first-row transition metals such as Cu has been predicted to convert bilayer MoS_2_ into a metallic state with simulated contact resistances as low as ~17–30 Ω·µm, approaching the quantum limit for a single conducting layer, although experimental verification of the ambient stability of transition-metal-intercalated MoS_2_ remains to be established [42].

Beyond chemical treatment, methods for inducing local phase transitions using external stimuli have also been studied. In particular, MoTe_2_ has a relatively small energy difference between its 2H and 1T′ phases, enabling local phase patterning; a low-resistance homojunction contact based on this approach has been experimentally demonstrated [43]. This phase-transition technology can be combined with defect engineering, strain engineering, and chemical doping within the material to enable more sophisticated device design [44,45,46].

Despite this progress, the practical deployment of phase engineering in a manufacturing environment still faces several open questions. Because the metallic 1T/1T′ phase is thermodynamically metastable relative to the 2H phase, the reverse transition back to the semiconducting phase can, in principle, be thermally activated; this raises reliability concerns during subsequent high-temperature process steps—such as dielectric deposition or contact annealing—that a fully integrated device flow would require [30,31,32]. In addition, most phase-transition demonstrations to date have been performed on individual flakes or small-area samples, and translating chemical or electrostatically induced phase conversion into a spatially selective, wafer-scale, and process-compatible module remains an important engineering task for realizing phase-engineered contacts as a mainstream contact-formation technique rather than a laboratory-scale proof of concept.

### 2.3. Metal/TMD Interface Properties and Contact Engineering

Because TMDs are layered materials with a low density of surface dangling bonds, it has been expected that, at an ideal interface, the Schottky barrier could be tuned according to the work function of the contacting metal. Early studies exploited this property by employing low-work-function Sc electrodes, reporting improved charge-injection characteristics in multilayer MoS_2_ FETs [47,48]. However, in practical metal/TMD interfaces, Fermi-level pinning can occur due to metal-induced gap states (MIGS), defects, and interfacial reactions, making it difficult to control the Schottky barrier solely through the metal work function [49,50]. In addition, interfacial damage and contamination generated during metal deposition can increase contact resistance [49,50,51].

Atomic-scale structural changes can also occur at real metal/TMD interfaces. Luo et al. observed Au_4_S_4_-based interfacial reconstruction at the interface between Au and monolayer MoS_2_ and reported that such structures can affect the band alignment and Schottky barrier at the interface [52,53]. Van der Waals (vdW) contact formation has been studied as an approach to reduce such non-ideal interfacial reactions and damage.

**Figure 5:**
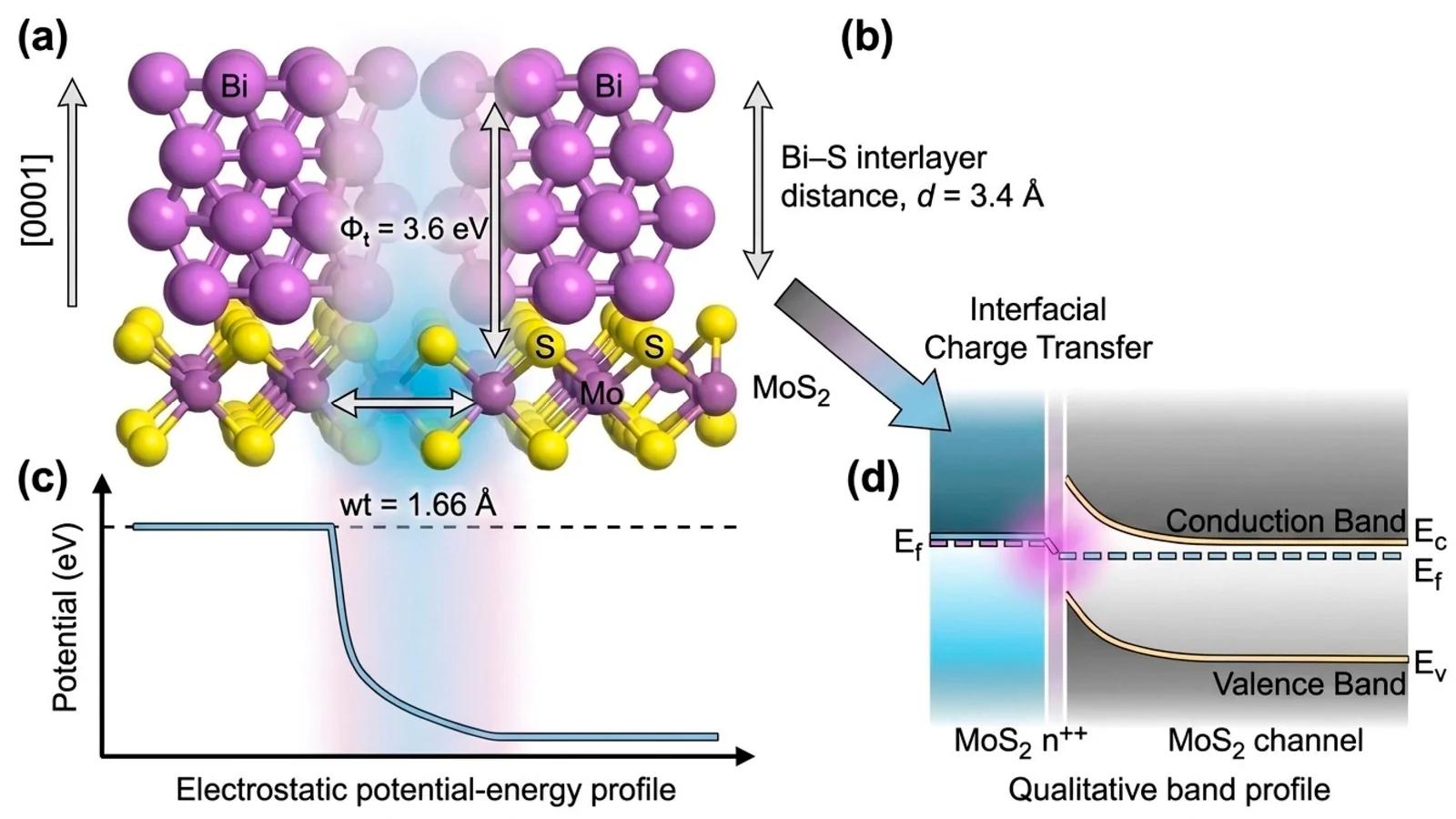
Schematic illustration of the Bi–MoS_2_ contact interface, concept adapted from [54]. (**a**) Simplified side-view ball-and-stick schematic of the interface, with the electron tunneling barrier region shaded (illustrative width wt = 1.66 Å, height Φt = 3.6 eV) and the Bi–S interlayer distance labeled (d = 3.4 Å). (**b**) Schematic illustration of the direction of interfacial charge transfer. (**c**) Illustrative, qualitative electrostatic potential-energy profile along the vertical direction. (**d**) Simplified, qualitative band profile of MoS_2_ near the contact.

More recently, non-destructive contact processes that minimize deposition energy have been pursued in order to preserve the intrinsic work function and band alignment of the material. A representative approach employs semimetal electrodes such as bismuth (Bi). At the Bi/MoS_2_ interface, the formation of metal-induced gap states (MIGS) is suppressed, enabling a low Schottky barrier and efficient electron injection, with a reported low contact resistance of approximately 123 Ω·μm [54]. The interfacial structure and charge-transfer behavior of the Bi/MoS_2_ contact are illustrated schematically in Figure 5.

Van der Waals (vdW) contact formation, in which the metal is bonded to the TMD surface via transfer rather than direct deposition, reduces the chemical reactions and lattice damage that can occur during deposition. This approach enables the formation of a clean interface, mitigating Fermi-level pinning and improving charge-injection characteristics [55,56]. In addition, vdW heterostructures employing h-BN and graphene have been widely studied as a means of protecting TMDs from external contamination and process-induced damage while maintaining interfacial quality [57,58,59,60,61,62]. Beyond improving the electrode and interfacial structure, contact resistance can also be reduced by doping the contact region. Increasing the carrier concentration in the contact region reduces the effective width of the Schottky barrier, facilitating charge injection. Reduced contact resistance has been reported in MoS_2_ and WS_2_ FETs using surface doping with benzyl viologen and chloride doping [63,64]. Substitutional doping with elements such as Nb has also been studied as a means of tuning the charge-transport properties and Fermi level of TMDs [65]. As described above, the contact resistance at metal/TMD interfaces can be controlled through various approaches, including electrode material selection, vdW contact formation, and carrier doping, making contact engineering a key process element for improving the performance of TMD-based devices.

Because the improvements reported for these contact strategies are often only a few Ω·µm apart, meaningful comparison between studies requires a consistent measurement methodology. Most of the contact resistance values discussed in this review, including the Bi/MoS_2_ result of approximately 123 Ω·µm [54], are extracted using the transfer length method (TLM), in which the total resistance of test structures with systematically varied channel lengths is measured and extrapolated to zero channel length to isolate the contact contribution from the intrinsic channel resistance. While TLM is well established for bulk semiconductors, its application to atomically thin, easily damaged TMD channels requires careful attention to probe-induced damage, channel-length-dependent doping non-uniformity, and the influence of the measurement contacts themselves on the extracted value, all of which can make reported contact resistance figures difficult to compare precisely across different laboratories and measurement conditions [51,53,54,56].

Table 2 summarizes representative contact-engineering strategies discussed above, together with their reported outcomes. Taken together, these results indicate a clear trend: the most effective approaches are those that avoid direct, energetic deposition of metal onto the pristine TMD surface, whether by substituting a semimetal with an intrinsically low density of states near the Fermi level, by transferring a pre-formed metal film, or by locally converting the TMD itself into a metallic phase. This shared principle—minimizing chemically and structurally disruptive processing at the contact interface—provides a useful design rule for future contact development, even though a single approach that simultaneously satisfies low resistance, wafer-scale manufacturability, and thermal-budget compatibility with back-end-of-line processing has not yet been established.

### 2.4. Chemical Vapor Deposition (CVD) and MOCVD

One of the key challenges for the practical application of TMDs is the uniform, wafer-scale growth of high-quality thin films. Mechanical exfoliation is useful for obtaining highly crystalline TMD flakes, but it offers limited control over flake position and size and has low throughput, making it unsuitable for direct application in large-area semiconductor manufacturing. Accordingly, large-area TMD growth techniques based on CVD and MOCVD have been actively studied [66]. Early CVD studies proposed methods for growing large-area MoS_2_ atomic layers using precursors such as MoO_3_ and sulfur [11], and subsequent research advanced toward synthesizing uniform, centimeter-scale monolayer and few-layer films by controlling growth conditions and precursor supply [67,68]. However, when MoS_2_ crystal domains nucleated at different locations merge during CVD growth, grain boundaries can form due to differences in crystal orientation. Such grain boundaries can degrade the electrical and optical properties of TMD thin films [68,69,70]. To reduce this effect, epitaxial growth techniques that control the growth orientation of TMDs on crystalline substrates such as sapphire have been studied [71].

**Figure 6:**
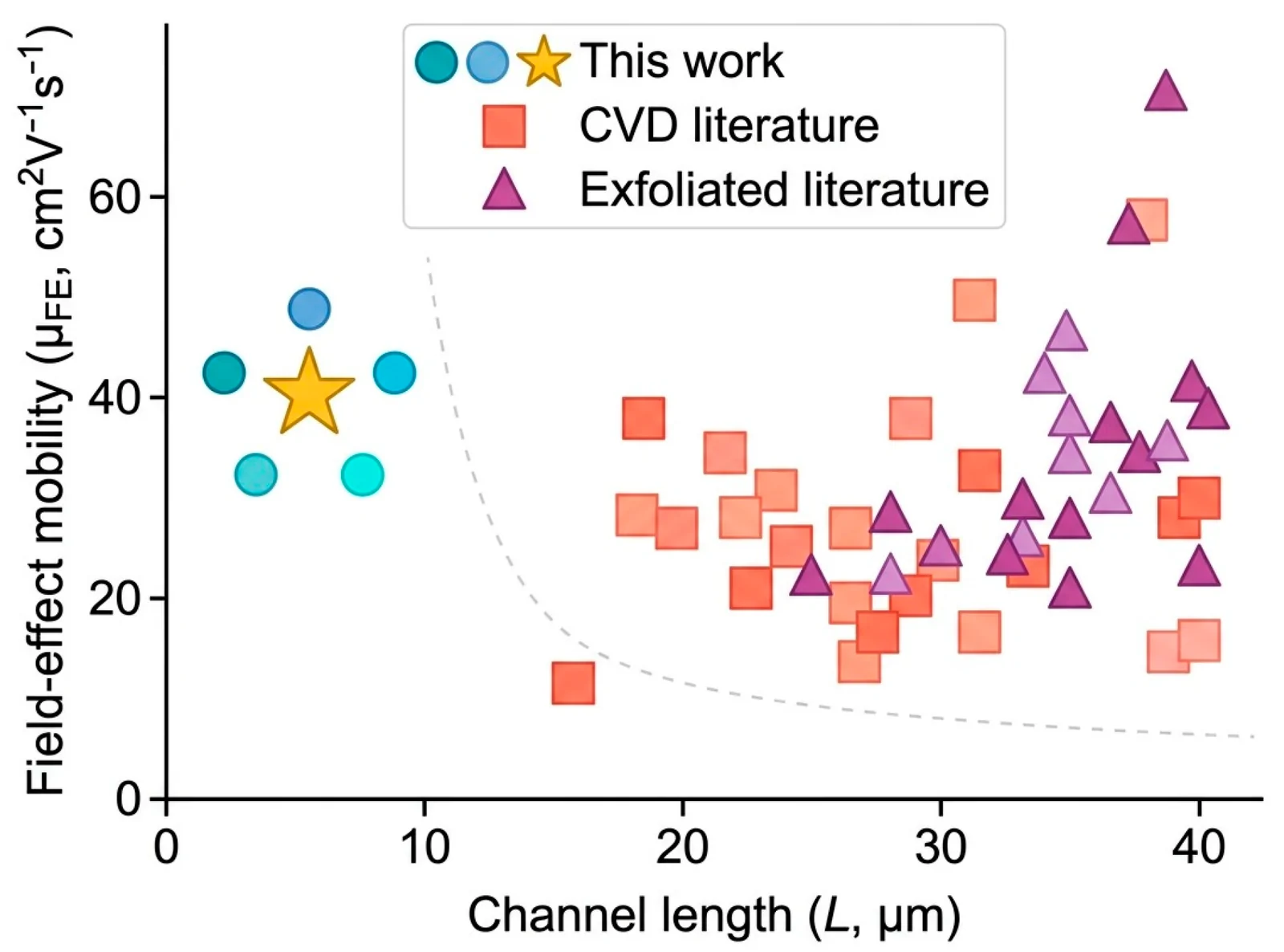
Illustrative schematic comparison of field-effect mobility (μFE) versus channel length (L), showing the qualitative trend in this work (five devices, circles; median as star), CVD-grown samples from the literature (squares), and exfoliated samples from the literature (triangles), concept adapted from [75].

MOCVD enables precise control over the supply rate and composition ratio of gas-phase precursors, offering advantages in process reproducibility and large-area uniformity [72,73,74]. Kang et al. used MOCVD to grow MoS_2_ and WS_2_ thin films with over 99% layer uniformity on 4-inch wafers, and reported a field-effect mobility of approximately 30 cm^2^ V^−1^ s^−1^ in FETs fabricated from the resulting films [75]. As illustrated schematically in Figure 6, the field-effect mobility of these MOCVD-grown devices is compared as a function of channel length with literature values for CVD-grown and exfoliated samples [75]. In MOCVD growth, nucleation density and precursor supply conditions also have a significant influence on crystal size and layer number. Methods for growing large monolayer MoS_2_ domains through nucleation control using alkali-metal halides have been reported [76], and research has also been conducted on forming uniform, wafer-scale MoS_2_ films by tuning process parameters such as precursor supply ratio and growth temperature [12,77].

Typical MOCVD routes to MoS_2_ and WS_2_ employ a volatile metal–organic or metal-carbonyl transition-metal precursor (e.g., Mo(CO)_6_ or W(CO)_6_) together with a gas-phase or organosulfur chalcogen source (e.g., H_2_S or an organosulfide such as diethyl sulfide), co-delivered in an Ar/H_2_ carrier stream into a hot-wall or showerhead reactor. Because both precursors decompose and react at the growth surface rather than being pre-reacted in the gas phase (in contrast to some powder-source CVD approaches), the precursor flow ratio, partial pressure, and substrate temperature directly set the nucleation density and hence the resulting grain size, which is the principal factor governing large-area film uniformity, as summarized in Table 3.

**Figure 7:**
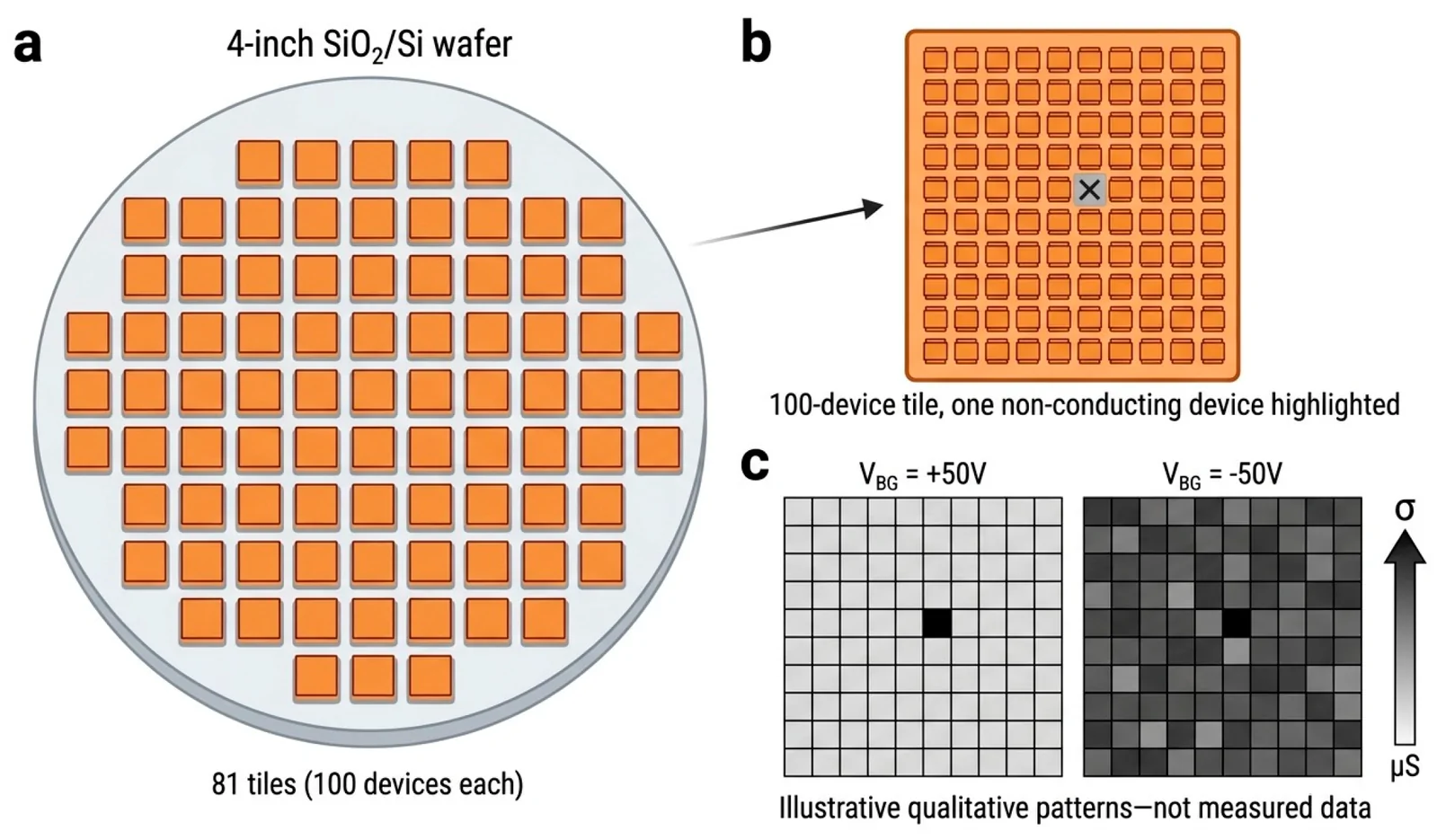
Schematic illustration of the batch-fabricated array of 8100 MoS_2_ FET devices on a 4-inch SiO_2_/Si wafer, concept adapted from [75]. (**a**) Schematic wafer layout (81 tiles of 100 devices each). (**b**) Schematic 100-device tile, with one device marked as non-conducting. (**c**) Illustrative conductivity color-map patterns at back-gate bias VBG = +50 V and −50 V.

We also note that the “>99%” figure reported below quantifies layer-number uniformity—i.e., the fraction of the wafer covered by the intended number of MoS_2_/WS_2_ layers—rather than electrical uniformity; layer-number uniformity, crystal-orientation uniformity, defect-density uniformity, and device-to-device electrical uniformity are distinct quantities that are not necessarily correlated, as illustrated by the spatial variation observed in the conductivity mapping of the 8100-device array grown by the same process (Figure 7; discussed further in Section 3.1).

These precursor-control techniques have also been applied to W-based TMDs in addition to MoS_2_, with reports of large-domain monolayer WSe_2_ and WS_2_ growth using alkali-metal halides [78,79]. In follow-up MOCVD studies, it was shown that controlling process parameters such as precursor pre-flow, precursor ratio, and growth temperature enables control over the 2D growth mode and layer number of MoS_2_, allowing the formation of homogeneous wafer-scale films [12,77].

MOCVD offers advantages in process reproducibility and large-area uniformity, but challenges such as carbon contamination, incomplete precursor decomposition, nucleation control, and high growth temperatures remain to be addressed in order to achieve high-quality TMD thin films [80]. In addition to large-area growth, epitaxial growth research aimed at controlling crystal orientation has also progressed. Li et al. achieved over 99% unidirectional domain alignment in 2-inch monolayer MoS_2_ by exploiting the step-edge structure of sapphire substrates [81]. This growth-orientation control technique has also been applied to WS_2_, with reports of 2-inch-scale single-crystal monolayer growth [82]. Furthermore, the atomic termination and symmetry of the sapphire surface have been identified as key factors determining the growth orientation of MoS_2_ [83]. More recently, TMD growth has been scaled up to the 12-inch level, and research continues to secure large-area uniformity and compatibility with existing semiconductor processes [84].

As summarized in Table 3, wafer-scale TMD growth has progressed rapidly from centimeter-scale polycrystalline films toward 12-inch wafers with device-relevant uniformity, broadly mirroring the trajectory of thin-film technologies that preceded it. A recurring theme across these studies is that uniformity and crystal quality are governed less by the deposition method itself than by control over nucleation—through substrate surface chemistry, step-edge templating, or nucleation-suppressing additives such as alkali-metal halides—since a high, randomly distributed nucleation density is the principal source of the grain boundaries that ultimately limit carrier mobility and device-to-device uniformity. This suggests that continued progress in nucleation control, rather than reactor engineering alone, is likely to be the key enabler for further improving wafer-scale TMD film quality.

**Table 3:** Representative large-area TMD growth studies by CVD and MOCVD.

Method	Material	Scale/Uniformity	Reported Mobility	Ref.
CVD (MoO_3_ + S precursors)	MoS_2_	Early large-area atomic layers	—	[11]
MOCVD	MoS_2_, WS_2_	4-inch wafer, >99% layer uniformity	≈30 cm^2^ V^−1^ s^−1^	[75]
MOCVD with alkali-metal-halide nucleation control	MoS_2_	Large monolayer domains	—	[76]
Sapphire step-edge epitaxy	MoS_2_	2-inch, >99% unidirectional alignment	—	[81]
a-plane sapphire epitaxy	WS_2_	2-inch single-crystal monolayer	—	[82]
Scaled epitaxial/MOCVD growth	MoS_2_	12-inch wafer	—	[84]

### 2.5. TMD-Based Ultrascaled Transistors and Next-Generation Device Applications

**Figure 8:**
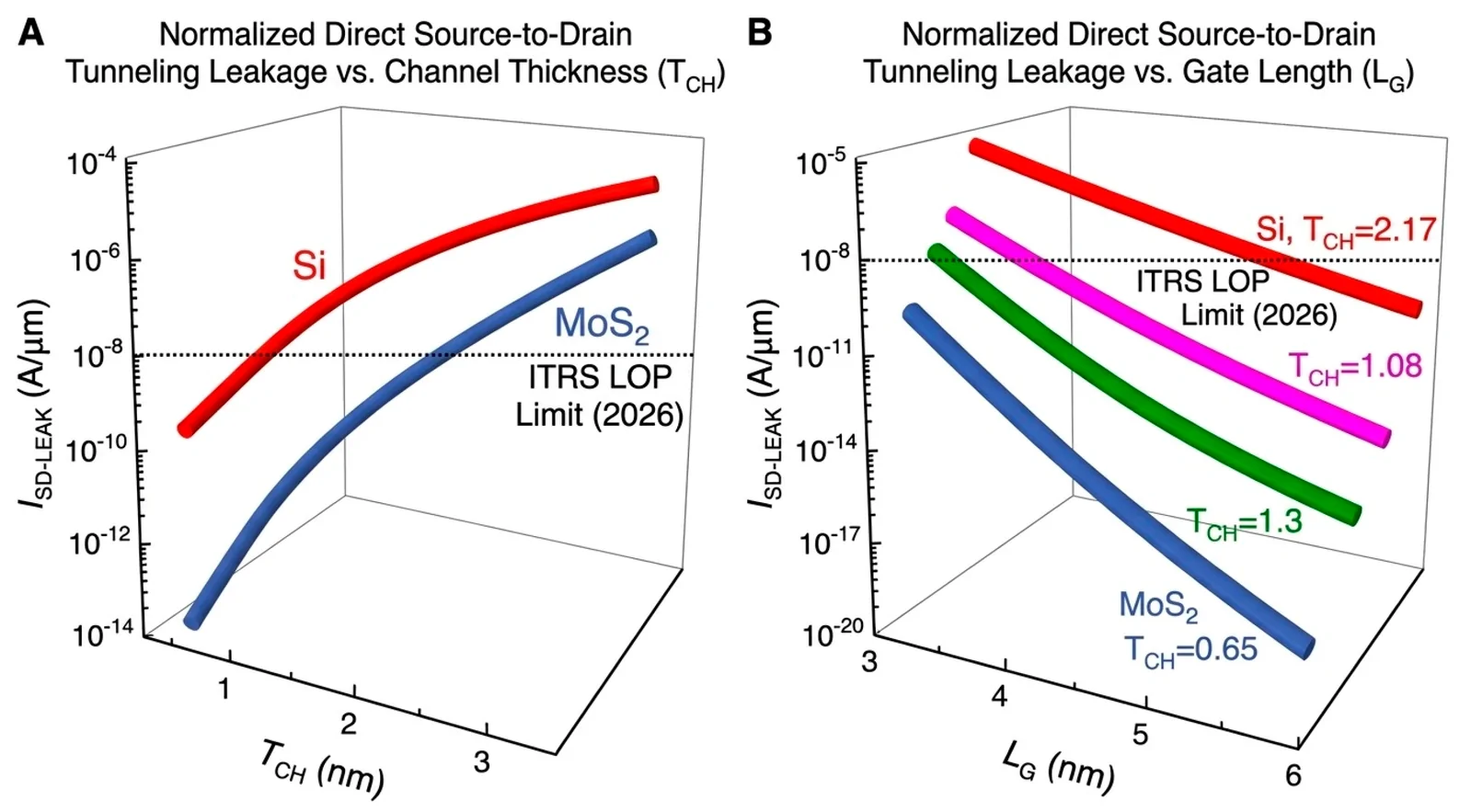
Illustrative schematic comparison of normalized direct source-to-drain tunneling leakage current for Si and MoS_2_ in the off state, concept adapted from [13]. (**A**) Qualitative trend versus channel thickness TCH. (**B**) Qualitative trend versus gate length LG. The dotted line in (**A**,**B**) indicates the illustrative low-operating-power (LOP) limit specified by the ITRS for the 2026 technology node.

Because of their atomically thin channel thickness, TMD semiconductors enable excellent electrostatic gate control and can suppress the short-channel effects that accompany device miniaturization, making them a next-generation channel material for extending the scaling limits of Si-based transistors [2,3,85,86]. Desai et al. demonstrated a MoS_2_ FET with a physical gate length of approximately 1 nm using a single-walled carbon nanotube (SWCNT) as the gate electrode [13]. This device exhibited a high on/off current ratio and excellent subthreshold characteristics even at this ultrascaled gate length, demonstrating the extreme scaling potential of TMDs [13,87]. This scaling potential has also been investigated for WS_2_ in addition to MoS_2_, with analyses of the electrical characteristics and short-channel effects at short channel lengths [88]. The off-state direct source-to-drain tunneling leakage current of Si and MoS_2_ channels is compared qualitatively as a function of channel thickness and gate length in Figure 8 [13].

In particular, atomically thin channels are advantageous for suppressing short-channel effects such as drain-induced barrier lowering (DIBL) [2,3]. Subsequently, Wu et al. further extended the extreme scaling potential of TMD transistors by demonstrating a sub-1 nm physical gate length in a vertical MoS_2_ transistor using an atomically thin graphene edge as the gate electrode [14]. However, as device dimensions shrink, contact resistance accounts for an increasingly larger fraction of the total device resistance; therefore, achieving extreme scaling requires not only electrostatic control of the channel but also improvements in contact resistance [53,55,56,59,89].

It is important to note that the physical gate length demonstrated in these studies is not equivalent to the channel length or to the contact (source–drain) pitch of a manufacturable device. Physical gate length refers specifically to the metallurgical length of the gate electrode; the effective channel length can differ from it depending on the source/drain access geometry, and the contact pitch—the parameter that most directly determines achievable device density in a circuit—is set by the combined footprint of the contact metal, isolation spacers, and lithographic alignment tolerance, none of which has necessarily been scaled in step with a given gate length. A recent wafer-scale demonstration makes this distinction concrete: a 2-inch monolayer-MoS_2_ process achieving a 0.34 nm physical gate length nonetheless reports a co-scaled channel length of 10 nm and a lateral contact pitch of approximately 60 nm, together with fully integrated logic gates (inverters, NAND, NOR) [90]; a separate all-2D vertical device achieves an 8–10 nm channel length with a transfer/contact length below 14 nm and a vertical device pitch below 30 nm [91]. Realizing ultrascaled gate lengths as a manufacturable technology node therefore additionally requires progress in on-state drive-current density, parasitic contact and access resistance, gate-dielectric reliability at the corresponding equivalent oxide thickness, device-to-device variability, and integration with a scaled interconnect stack—challenges that are distinct from, and in several respects more difficult than, demonstrating the gate length in isolation.

The application of TMD FETs is expanding beyond digital logic to high-frequency devices. GHz-range cutoff frequencies have been experimentally reported for MoS_2_-based transistors [92,93,94,95,96], and further reductions in channel length and contact resistance are expected to extend operation to even higher frequency ranges. The integration-density improvement enabled by TMDs is not limited to reducing the lateral dimensions of transistors. Because of their atomic thinness, TMDs have also been proposed for monolithic 3D integration, in which additional device layers are formed on top of Si CMOS [97,98]. Complementary transistor structures vertically integrating n-type and p-type 2D semiconductors have also been demonstrated, representing a new approach to increasing integration density using 2D materials [99,100]. However, realizing practical CMOS integration of TMDs requires the simultaneous resolution of several challenges, including contact resistance, wafer-scale material uniformity, dielectric integration, defect control, and compatibility with existing processes [62,101,102,103,104].

Table 4 collects representative ultrascaled TMD transistor demonstrations discussed in this section. A consistent finding across these reports is that the electrostatic benefit of an atomically thin channel is realized primarily in the subthreshold regime—that is, in a steep subthreshold swing and a high on/off current ratio—rather than in a proportional increase in on-state drive current, since the latter is increasingly limited by contact resistance rather than by the intrinsic channel itself as the gate length is reduced. This observation reinforces the conclusion, echoed throughout Section 2.3, Section 2.4 and Section 2.5, that contact engineering and channel-length scaling must advance together: a transistor with an excellent sub-1 nm gate length but an unoptimized contact will fail to translate its electrostatic advantage into a competitive drive current.

## 3. Challenges and Future Outlook for Industrial Integration

The results reviewed in Section 2 demonstrate that TMD-based devices have advanced rapidly at the level of individual demonstrations—wafer-scale films, ultralow-resistance contacts, and sub-1 nm gate-length transistors have each been achieved separately. However, moving from these isolated proof-of-concept results to a manufacturable technology requires that material quality, contact resistance, dielectric integration, and process compatibility be solved simultaneously and reproducibly across an entire wafer. This section discusses four such cross-cutting challenges and the research directions being pursued to address them.

### 3.1. Wafer-Scale Material Uniformity, Defect Density, and Metrology

Although MOCVD and epitaxial growth techniques have achieved layer uniformity exceeding 99% and wafer diameters up to 12 inches [75,84], uniformity in layer number does not, by itself, guarantee uniformity in electrical performance. Grain boundaries formed when independently nucleated domains merge [68,69,70], point defects such as chalcogen vacancies, and unintentional doping from the growth or transfer process can all vary spatially across a wafer even when the film appears uniform by optical or layer-count metrics. The batch fabrication of 8100 MoS_2_ FETs on a single 4-inch wafer illustrates both the promise and the difficulty of this challenge: the vast majority of devices were functional, yet spatial maps of device conductivity still revealed measurable variation across the wafer [75]. Realizing the device-to-device and wafer-to-wafer uniformity required for integrated circuits will likely require the semiconductor industry’s standard toolkit—statistical process control, in-line optical and electrical metrology, and defect-density specifications—to be adapted to the specific defect types and length scales relevant to 2D materials, an effort that is still in a comparatively early stage relative to established Si process control [70,73,80].

The optical and structural characterization techniques discussed earlier in this review as tools for fundamental TMD research—photoluminescence mapping to track layer-number and strain variation, Raman spectroscopy to monitor the E12g/A1g mode splitting associated with layer number and doping, and atomic-resolution STEM to directly image grain boundaries and coordination changes [6,25,26,34,35]—are also the most practical candidates for wafer-level, non-destructive metrology, since each can, in principle, be automated into a mapping tool that scans an entire wafer rather than a single flake. Adapting these techniques from single-point, publication-oriented measurements into fast, spatially resolved, statistically meaningful metrology protocols, with defect-density and uniformity specifications analogous to those used for Si wafers, represents a comparatively underexplored but practically essential step on the path toward manufacturable TMD material supply.

### 3.2. Approaching the Contact-Resistance Quantum Limit

Contact resistance has featured prominently throughout this review because it increasingly dominates total device resistance as the channel length is scaled down [53,55,56,59,89]. Recent work employing van der Waals and semimetal contacts has reported contact resistance values approaching the theoretical quantum limit set by the number of available conduction channels in a monolayer semiconductor [54,56], indicating that the fundamental physical barriers to low-resistance contacts are becoming increasingly well understood. The remaining challenge is therefore less about discovering new contact physics and more about engineering: semimetal deposition and vdW transfer processes that have so far been demonstrated on small, individually assembled samples must be adapted into wafer-scale, high-throughput, and thermally compatible process modules before their benefits can be realized in manufactured circuits [54,55,56]. Bridging this gap between champion laboratory results and reproducible, wafer-scale contact modules is likely to be one of the most consequential near-term challenges for TMD device technology.

Quantitatively, the width-normalized quantum (ballistic) contact resistance follows Rcᵀ·W = (h/2 × 10^2^)·(1/Meff), where h/2 × 10^2^ is the resistance quantum (which already incorporates the spin degeneracy gs = 2) and Meff is the effective number of conducting modes set by the contact-region carrier density n2D and the valley degeneracy gv of the contacted 2D channel; because Meff scales as $\sqrt{n2D}$, the quantum limit itself is carrier-density-dependent rather than a single fixed number. For monolayer MoS_2_ (gv = 2), this reduces to Rcᵀ·W ≈ 36/$\sqrt{n2D}$ Ω·µm (n2D in units of 10^13^ cm^−2^), whereas a single-valley 2D semiconductor (gv = 1) has an intrinsically higher quantum-limited contact resistance, Rcᵀ·W ≈ 51/$\sqrt{n2D}$ Ω·µm [105]. Because the quantum limit depends on the contact-region carrier density—which is set by doping, gating, or the metallic-phase/semimetal contact configuration and is not always reported—contact-resistance values obtained under different carrier-density, temperature, or extraction-method (e.g., two-terminal vs. transfer-length-method vs. four-probe) conditions should not be compared directly without normalizing for these factors.

### 3.3. Gate Dielectric Integration and Device Reliability

The same absence of dangling bonds that gives TMDs their atomically smooth, low-defect-density surfaces also makes it intrinsically difficult to nucleate a uniform, pinhole-free high-k dielectric directly on the TMD surface, since conventional atomic layer deposition relies on reactive surface sites to initiate growth [73,101,102]. Early demonstrations of high-k-gated monolayer MoS_2_ FETs relied on nucleation layers or seed treatments to enable reasonable dielectric coverage [8], and continued refinement of low-damage, high-uniformity dielectric integration remains an active research area. Beyond the initial dielectric deposition, the long-term electrical reliability of TMD FETs under gate bias and thermal stress—including threshold-voltage stability, interface-trap density, and time-dependent dielectric breakdown—has received comparatively less systematic study than growth and contact engineering, even though these reliability characteristics are ultimately what determine whether a device technology can meet industrial qualification standards [73,89].

Quantifying these reliability characteristics generally requires electrical techniques adapted from conventional MOSFET characterization—capacitance–voltage (C–V) measurements to extract interface-trap density near the dielectric/channel interface, Hall-effect measurements to separate carrier density from mobility, and constant-voltage or constant-current stress tests to track threshold-voltage drift over time—applied to device structures that are far more sensitive to processing damage than their bulk-Si counterparts. Because the entire channel of a monolayer TMD device is, by definition, an interface, defects and traps that would be buried and comparatively benign in a bulk Si MOSFET can dominate the electrical behavior of a monolayer device, making reliability characterization simultaneously more important and more experimentally challenging for TMD FETs than for their conventional counterparts.

### 3.4. Process Compatibility, Scalability, and Manufacturing Considerations

Finally, integrating TMDs into existing semiconductor manufacturing lines requires more than material and device performance; the growth, transfer, and contact-formation steps must also be compatible with the thermal budgets, chemical purity requirements, and equipment infrastructure of a CMOS fab. This constraint is particularly acute for monolithic 3D integration, in which TMD device layers are formed directly on top of finished Si CMOS circuitry and must therefore be processed at temperatures low enough to avoid degrading the underlying transistors and interconnects [97,98]. Direct, transfer-free growth of TMDs at CMOS-compatible temperatures, together with the elimination of process steps that introduce contamination or require high-temperature annealing, is therefore an important direction for future process development [74,80]. Taken together with the roadmap perspectives offered elsewhere in the literature [15,86,103], the technical challenges summarized in this section suggest that the transition of TMDs from laboratory-scale demonstrations to manufacturable technology will likely proceed incrementally, application by application, beginning with contexts—such as back-end-of-line sensors, RF front ends, or specialty logic—where the unique advantages of atomically thin channels provide the clearest performance benefit relative to the integration cost, before more general-purpose CMOS logic applications become viable at scale.

For context, back-end-of-line (BEOL)-compatible thermal budgets in current CMOS practice are generally required to remain below approximately 400 °C for extended process steps, to avoid degrading underlying interconnect and device reliability; several of the growth and phase-engineering routes discussed in this review—including conventional CVD/MOCVD growth temperatures and the thermally activated 1T-to-2H reverse phase transition discussed in Section 2.2—currently exceed this budget, whereas recent low-temperature gate-dielectric integration work has demonstrated CMOS-compatible processing at 100–200 °C for the gate stack alone, achieving a 0.9–1.1 nm equivalent oxide thickness with subthreshold swing of 70–80 mV/dec and an on/off ratio exceeding 10^6^–10^7^ on monolayer MoS_2_ and WSe_2_ [106]. This illustrates that budget-compatible integration is achievable for individual process modules even though a fully thermal-budget-compatible growth-to-contact process flow has not yet been demonstrated end to end. Beyond thermal budget, a manufacturable process will additionally need to meet wafer-scale variability, contamination-control, and alignment-tolerance specifications broadly analogous to those used in Si CMOS—for example, the sub-30-to-60 nm contact/device pitch and gate-to-contact alignment already demonstrated in the wafer-scale and all-2D devices discussed in Section 2.5 [90,91]—together with standardized reliability qualification (e.g., time-dependent dielectric breakdown and bias-temperature-instability testing per Section 3.3) before TMD devices can be assessed against established CMOS manufacturing criteria. At present, few of the individual demonstrations reviewed here report data against all of these criteria simultaneously, which is itself the central roadmap gap identified in peer review.

### 3.5. Emerging Directions: Heterostructures and Beyond-CMOS Applications

Beyond incremental improvements to the individual process modules discussed above, several emerging directions may open additional application space for TMDs that does not require displacing Si logic outright. Van der Waals heterostructures, in which different 2D materials such as TMDs, h-BN, and graphene are stacked without the lattice-matching constraints that govern conventional epitaxy, allow band alignment and functionality to be engineered by choice of stacking sequence rather than by chemical synthesis alone, and have already been used to realize clean contacts and protected channels in this review [57,58,59,60,61,62]. Vertically stacked complementary n-type and p-type 2D transistors point toward compact logic and memory building blocks that exploit the thinness of TMDs for area scaling rather than channel-length scaling alone [99,100]. In parallel, the demonstrated GHz-range cutoff frequencies of TMD transistors suggest a role in radio-frequency front ends and flexible or transparent electronics, application spaces that place less demanding requirements on absolute mobility and integration density than high-performance digital logic while still benefiting from the mechanical flexibility and optical transparency that atomically thin channels can offer [92,93,94,95,96]. These emerging directions suggest that the near-term industrial value of TMDs may be realized as much through new device functionalities enabled by their two-dimensional form factor as through direct replacement of Si channels in conventional logic transistors (Table 5).

## 4. Conclusions

In this review, we examined the material properties of two-dimensional transition metal dichalcogenides (TMDs) and the key process technologies and research trends relevant to their application in next-generation semiconductor devices. TMDs exhibit atomically thin thicknesses and layer-dependent band-structure variation; in particular, the direct bandgap and excellent electrostatic gate control observed in monolayer MoS_2_ provide a foundation for their use as channel materials not only in electronic and optoelectronic devices but also in ultrascaled transistors.

Improving the performance of TMD-based devices requires not only optimizing the intrinsic properties of the material but also controlling the interface between the electrode and channel. The phase engineering approaches discussed in this review exploit differences in the electrical properties of crystal phases such as 2H, 1T, and 1T′ to tune the conduction characteristics of the contact region. In addition, contact-engineering techniques such as semimetal electrodes, van der Waals (vdW) contacts, and doping of the contact region have been proposed as key strategies for mitigating Fermi-level pinning and high contact resistance at metal/TMD interfaces.

In terms of large-area material growth, advances in CVD and MOCVD technology are enabling wafer-scale growth of TMD thin films, and research is ongoing to improve film uniformity and crystallinity by controlling nucleation and precursor-supply conditions. In particular, epitaxial growth techniques for reducing grain-boundary formation and controlling growth orientation represent an important research direction for realizing large-area single-crystal TMD films. However, challenges such as achieving uniform material properties across the entire wafer, controlling defects and contamination, and ensuring process reproducibility must continue to be addressed for practical semiconductor manufacturing applications.

At the device level, the experimental realization of 1 nm and sub-1 nm physical gate lengths using MoS_2_ has demonstrated the extreme scaling potential of TMDs. Furthermore, the application of TMD FETs is expanding beyond digital devices to GHz-range high-frequency devices and monolithic 3D integration. However, as device dimensions shrink, the relative impact of contact resistance increases, and dielectric interfaces, defect control, and compatibility with existing CMOS processes remain important challenges for practical integration.

Therefore, realizing TMDs as next-generation semiconductor materials will require, beyond simply improving individual device performance, the co-development of large-area high-quality material growth, low-resistance contact formation, stable interface and defect control, and integration technologies compatible with existing CMOS processes. If these process technologies continue to advance alongside effective utilization of the excellent electrostatic control enabled by the atomically thin structure of TMDs, TMDs are expected to serve as a promising next-generation channel material capable of complementing the scaling limitations of conventional Si-based semiconductors.

As discussed in Section 3, the path from the individually impressive results summarized in this review to a manufacturable TMD-based technology will require sustained, coordinated progress across wafer-scale metrology and defect control, contact resistance approaching its quantum limit under process-compatible conditions, reliable high-k dielectric integration, and thermal-budget-compatible process flows. No single result reviewed here resolves all of these requirements simultaneously, and we expect that near-term industrial adoption of TMDs is more likely to begin in application areas—such as radio-frequency front ends, sensing, or heterogeneously integrated back-end-of-line layers—that can exploit the unique advantages of atomically thin channels without demanding the full performance and reliability envelope of general-purpose CMOS logic. Continued cross-disciplinary progress in materials growth, device physics, and process integration will determine how quickly TMDs move from a promising channel material to a component of manufactured semiconductor technology.

## Figures and Tables

**Table 1:** Layer-dependent structural, optical, and electrical properties of MoS_2_ discussed in Section 2.1.

Property	Bulk	Monolayer	Ref.
Bandgap type	Indirect	Direct	[6,16,17,18]
Bandgap energy	≈1.29 eV	≈1.8–1.9 eV	[6,16,17,18]
A-exciton PL peak	Weak/indirect	Strong, ≈1.9 eV	[5,6]
Trion binding energy	—	≈20 meV	[20]
Exciton binding energy (WS_2_ monolayer)	—	≈0.32 eV	[21]
Strain coefficient (optical bandgap)	—	≈45 meV per % uniaxial strain	[23,24]
Theoretical phonon-limited mobility	—	≈410 cm^2^ V^−1^ s^−1^	[27,28]
Reported FET mobility (high-k gated)	—	>200 cm^2^ V^−1^ s^−1^	[8]
Carrier mobility (Hall, in-plane, 300 K)	On the order of 10–10^2^ cm^2^ V^−1^ s^−1^ in typical 2H-MoS_2_ crystals (phonon-limited; rises sharply at low T)	~200 cm^2^ V^−1^ s^−1^ (exp., high-k-gated FET); ~410 cm^2^ V^−1^ s^−1^ (theor., phonon limit)	[8,20,28] new

**Table 2:** Summary of representative contact-engineering strategies for metal/TMD interfaces.

Strategy	Representative System	Key Reported Outcome	Ref.
Low-work-function metal electrode	Sc/multilayer MoS_2_	Improved charge injection via reduced Schottky barrier	[47,48]
Semimetal (vdW) electrode	Bi/monolayer MoS_2_	Suppressed MIGS; contact resistance ≈ 123 Ω·µm	[54]
Van der Waals transferred contact	Transferred metal/MoS_2_	Clean interface; mitigated Fermi-level pinning	[55,56]
h-BN/graphene encapsulation	vdW heterostructure	Protection from contamination and process-induced damage	[57,58,59,60,61,62]
Surface charge-transfer doping	Benzyl viologen, chloride/MoS_2_, WS_2_	Reduced contact resistance via increased carrier concentration	[63,64]
Substitutional doping	Nb/TMD	Tuned Fermi level and carrier-transport properties	[65]
Phase-engineered (2H/1T) homojunction	Li-intercalated MoS_2_	Metallic contact region reduces contact resistance	[10,30,32,36,37,38,39,40]
Transition-metal (Cu) intercalation	Cu/bilayer MoS_2_ (computational, DFT/NEGF)	Predicted metallization; simulated contact resistance ≈ 16.7 (zigzag)/30.0 (armchair) Ω·µm, approaching the quantum limit	[42]

**Table 4:** Representative ultrascaled TMD transistor demonstrations.

Gate Architecture	Physical Gate Length	Channel Material	Key Reported Result	Ref.
SWCNT side-gate	≈1 nm	MoS_2_	High on/off ratio; steep subthreshold swing at LG = 1 nm	[13]
Graphene-edge vertical gate	Sub-1 nm	MoS_2_	Extended extreme-scaling limit below 1 nm	[14]
Planar top-gate (thickness-dependent study)	Short-channel scaling study	WS_2_	Thickness-dependent short-channel-effect analysis	[88]
Self-aligned angstrom-gate (wafer-scale, new)	0.34 nm (channel length 10 nm)	MoS_2_	2-inch wafer with integrated logic (inverter, NAND, NOR); Ion 23 → 172 µA/µm, SS 412 → 127 mV/dec, Ion/Ioff > 10^6^, contact pitch ≈ 60 nm, EOT 0.7 nm	[90]
All-2D vertical MS-FET (new)	n/a (vertical); channel length ≈ 8–10 nm	MoS_2_ (TiS_2_ semimetal gate, graphene S/D)	Ion ≈ 144 µA/µm @ 1 V, SS ≈ 62 mV/dec, Ion/Ioff > 10^7^, transfer/contact length < 14 nm, vertical pitch < 30 nm	[91]

**Table 5:** Summary of key challenges and candidate solutions for the industrial integration of TMD-based devices.

Challenge	Current Status (=Current Best Demonstration)	Candidate Approaches (→ Representative Manufacturing Requirement)	Ref.
Wafer-scale uniformity & defect density	>99% layer uniformity demonstrated on 4- to 12-inch wafers; grain boundaries remain	Nucleation control, step-edge epitaxy, standardized metrology Requirement: die-level yield and statistical process control comparable to Si wafer specifications; standardized wafer-mapping metrology.	[68,70,75,80,81,84]
Contact resistance	Approaching the theoretical quantum limit in laboratory demonstrations	Semimetal/vdW contacts adapted to wafer-scale processes Requirement: wafer-scale, high-throughput, thermally compatible contact module reproducing champion values across a full wafer; see also simulated 17–30 Ω·µm for Cu-intercalated bilayer MoS_2_ [42].	[54,56,89]
Gate dielectric integration	High-k nucleation on a dangling-bond-free surface remains difficult	Seed/nucleation layers; refined ALD process development Requirement: pinhole-free, reproducible sub-1 nm EOT across a full wafer with qualified reliability; 0.7–1.1 nm EOT now demonstrated at 100–200 °C [90,106].	[8,73,101,102]
Device reliability	Systematic bias- and thermal-stress data remain limited	Long-term reliability studies; standardized test protocols Requirement: standardized TDDB/BTI qualification protocols analogous to Si CMOS.	[73,89]
CMOS/3D process compatibility	Typical growth temperatures often exceed back-end-of-line thermal budgets	Direct, transfer-free, low-temperature growth Requirement: direct, transfer-free, low-temperature (≲400 °C) growth-to-contact flow.	[74,80,97,98]
Gate length/channel length/contact pitch co-scaling	0.34 nm gate length with 10 nm channel length and ~60 nm contact pitch, wafer-scale with logic gates [90]; 8–10 nm channel length, <30 nm vertical pitch [91]	Contact pitch and channel length scaled in step with gate length at a manufacturable node; circuit-level (not device-level) demonstration.	[13,14,90,91]

## Data Availability

No new data were created or analyzed in this study. Data sharing is not applicable to this article.
